# Integrated biosorption and bioreduction of hexavalent chromium using *Fusarium* species under optimized process parameters

**DOI:** 10.1038/s41598-026-61430-9

**Published:** 2026-07-15

**Authors:** Nora N. El-Gamal, Latifa A. Mohamed, Shimaa R. Hamed, Magda A. El-Bendary

**Affiliations:** 1 Microbial Chemistry Department, Biotechnology Research Institute, National Research Centre, 33 Bohouth Street, Dokki, Giza, Egypt; 2 Microbial Biotechnology Department, Biotechnology Research Institute, National Research Centre, Bohouth Street, Dokki, Giza, Egypt

**Keywords:** Bioremediation, Biosorption, Chromate reductase, *Fusarium*, Biological techniques, Biotechnology, Environmental sciences, Microbiology

## Abstract

Fungal biosorption and bioreduction of hexavalent chromium (Cr(VI)) provide a sustainable and cost-effective alternative to traditional physicochemical methods by utilizing microbial enzymatic pathways for detoxification. *Fusarium verticillioides* and *Fusarium oxysporum* were isolated and tested for their ability to remove Cr(VI) from aqueous solutions. Biomass and filtrate obtained after growth of *F. verticillioides* at pH 2 showed a maximum Cr(VI) removal achieving 77% and 79% removal, respectively.While biomass and filtrates obtained after growth of *F. oxysporum* at pH4 showed the optimal Cr(VI) removal (69%) for biomass and (97%) for filtrate, respectively. Increasing inoculum size up to 38 × 10^8^ CFU enhanced Cr(VI) reduction efficiency, but removal decreased with higher Cr(VI) concentrations, with no removal observed at 60 mg/l. Removal efficiency also increased significantly with incubation time up to five days. Scanning electron microscopy revealed structural changes in fungal biomass associated with biosorption and reduction of Cr(VI) to the less toxic Cr(III), supported by detection of chromate reductase activity in fungal exo- and endo-filtrates as well as membrane debris. Atomic absorption spectroscopy indicated that *F. verticillioides* and *F. oxysporum* converted approximately 95% and 78% of Cr(VI) to Cr(III), respectively, suggesting these fungi offer eco-friendly bioremediation strategies by combining cell-wall biosorption with enzymatic bioreduction mechanisms to transform toxic chromium into less harmful forms.

## Introduction

Humans and animals may suffer heavy metal toxicity from contaminated sources by extended exposure through inhalation, food consumption, and skin contact^1^. Hexavalent chromium (Cr(VI)) is a highly toxic and carcinogenic heavy metal widely used in industries such as electroplating, tanning, metal finishing, petroleum refining, wood processing, nuclear power plants, ceramic, textile, glass, auto components, pigments, etc., posing significant environmental and health risks through contamination of soil and water^2–4^. Chromium exists mainly in two stable forms: the less toxic trivalent Cr(III), which is essential for mammalian metabolism such as glucose, lipid, and protein metabolism, and the more hazardous Cr(VI), which is prioritized for removal due to its mutagenicity, carcinogenicity and mobility^1,5,6^. Additionally, the ecosystem and its biota are negatively impacted by Cr(VI).

Conventional physico-chemical methods for Cr(VI) removal including membrane filtration, chemical precipitation, ion-exchange, biosorption, oxidation, coagulation, reduction, electrochemical reactions, reverse osmosis, photocatalysis, nanotechnology, etc., often face challenges like high large-scale application, costs, energy consumption, incomplete metal removal, and secondary pollution, prompting interest in biological remediation approaches^7,8^.

Microorganisms including bacteria and fungi can reduce Cr(VI) to Cr(III) via enzymatic bioreduction while also adsorbing chromium through biosorption mechanisms involving cell surface functional groups, making bioremediation a sustainable and cost-effective alternative^9,10^.

Fungi have demonstrated particularly high efficiency in chromium biosorption and reduction, with recent studies identifying specific reductases and cell wall components that facilitate these processes. Fungi, bacteria, and other microorganisms have been found to exhibit a variety of cellular responses for heavy metal-induced stress tolerance, including oxidation–reduction reactions, biosorption by cell biomass, bioaccumulation, binding by cytosolic molecules, and protein DNA adduct formation^11^. Overall, microbial bioremediation combines biosorption, bioreduction, and biomineralization to detoxify Cr(VI), offering promising potential for sustainable large-scale environmental applications with minimal secondary contamination^12^. The processes of biosorption and bioreduction work in concert to bioremediate Cr(VI). Microorganisms can directly or indirectly reduce Cr(VI) to Cr(III) both inside and outside of cells due to the reductase that cells produce. On the surface of microbial cells, some functional groups including hydroxyl, carboxyl, and amino interact with Cr(VI) or Cr(III) to create complexes. While Cr(III) and Cr(VI) can both be accumulated intracellularly, Cr(VI) is rapidly reduced to the less toxic Cr(III) after entering microbial cells, which then tends to bind to cellular components^13^. Microorganisms not only absorb heavy metals but also convert the more toxic forms of metals into less dangerous ones through their oxido-reduction enzymatic processes^14^.

Cr(VI) biosorbents have been produced from fungi, bacteria, algae, plants, extracted biological materials, and modified biomaterials^15^. Compared to other biological sources, the fungi demonstrated superior heavy metal biosorption and accumulation efficiency. Furthermore, oxidation–reduction processes are the metabolic responses that fungi compete chromium stress^11^. Numerous studies have documented how microorganisms, including bacteria (*Bacillus sp*., *Exiguobacterium sp*., *Staphylococcus aureus*, *Pediococcus pentosaceus*, *Stenotrophomonas maltophilia*, *Pantoea sp*., and *Aeromonas sp*.) and fungi (*F. proliferatum*, *Candida utilis*, *Candida maltosa*, *Hypocrea tawa*, *Paecilomyces lilacinus*, *Aspergillus parasiticus*, *Aspergillus niger*, and *Pichia* sp.) may utilize and metabolically change Cr(VI) into the less toxic Cr(III)^16,17^. Recent work by Chatterjee and Das^18^ identified NADH-dependent chromate reductases in *Aspergillus niger* that catalyze extracellular electron transfer, reducing Cr(VI) to Cr(III) via intermediate radicals (Cr(V)/Cr(IV)). Similarly, Wang et al.^19^ reported that fungal cell wall components (chitin, glucans) provide binding sites for Cr(VI) biosorption, followed by enzymatic reduction mediated by glutathione reductase and cytochrome P450 systems.

This study aimed to isolate and identify chromium-tolerant fungi and to assess their capacity for Cr(VI) detoxification. The specific objectives were to optimize the culture conditions influencing chromium removal, compare the contributions of fungal biomass and culture filtrate, and investigate the involvement of chromate biosorption and bioreduction.

## Material and methods

**Fig. 1:**
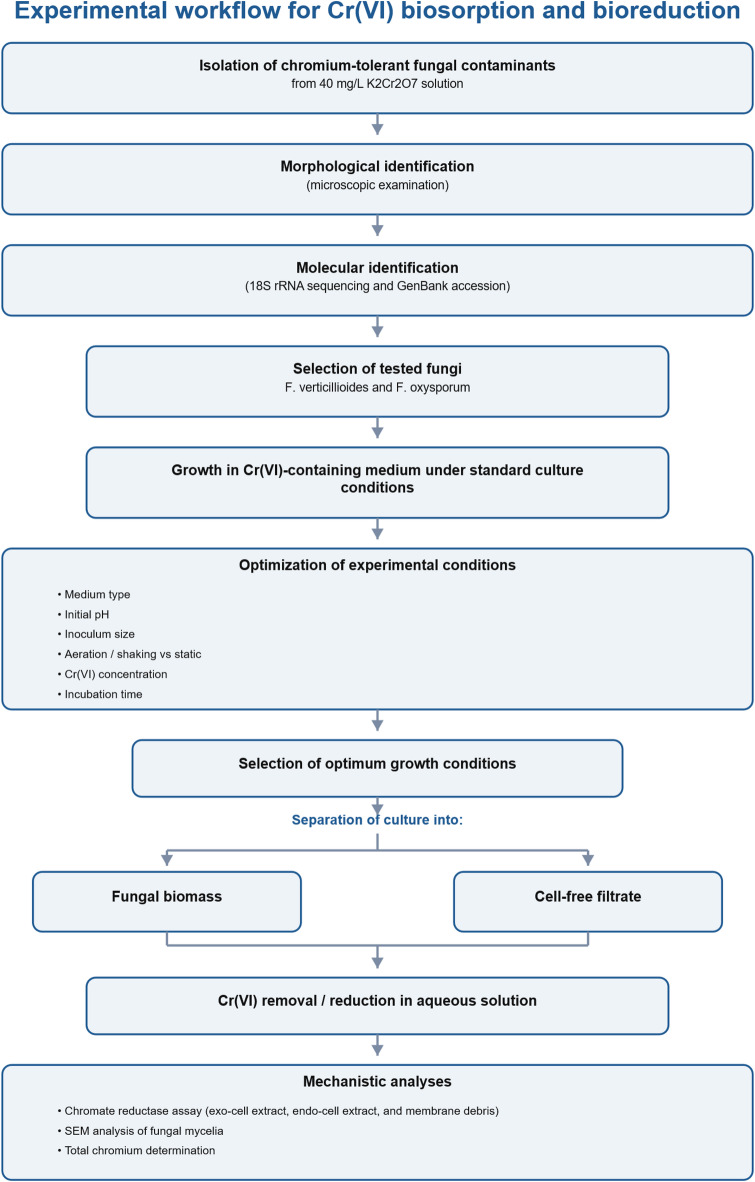
The experimental workflow used in this study for biosorption and bioreduction of hexavalent chromium using *Fusarium* Species.

The overall experimental workflow used in this study is summarized in Fig. 1.

### Chemicals

Potassium dichromate (K_2_Cr_2_O_7_) with a purity of 95% and 1, 5-diphenyl-carbazide were purchased from Merck Co., Germany. High analytical grade chemicals were also present.

### Microorganisms

The two fungal cultures used in this study were isolated as contaminants from a flask containing 40 mg K_2_Cr_2_O_7_/l. They were morphologically and molecularly identified and deposited in the GenBank under the accession numbers of ON100819 and ON010524.

### Growth media and Cr(VI) preparation

Malt Extract Broth: 20 g/l malt extract, 20 g/l dextrose, 6 g/l peptone.

Potato Dextrose Broth: Prepared per manufacturer’s instructions (HiMedia).

Czapek Dox Broth: 30 g/l glucose, 2 g/l NaNO₃, 1 g/l KH₂PO₄, 0.5 g/l MgSO₄, 0.5 g/l KCl.

Sabouraud’s Dextrose Broth: 40 g/l dextrose, 10 g/l peptone.

Cr(VI) stock solution: 1 g/l K₂Cr₂O₇ (Merck, Germany) in distilled water, filter-sterilized (0.22 μm).

For preparation of solid media: 15 g/l agar was added.

## Methods

### Isolation of microorganisms

During the preparation of a 40 mg/l chromate solution in tap water, weak microbial growth was observed in one flask lift for 21 days at room temperature, prompting isolation of organisms from this solution. Two fungal cultures were isolated and sub-cultured on Czapek Dox agar containing 5 mg/l K_2_Cr_2_O_7_, then incubated at 28 °C for 5 days; pure cultures were maintained on malt extract agar slants at 4 °C.

### Identification of isolated fungi

#### Morphological identification

Morphological features such as colony diameter, conidia color, extracellular exudates, pigmentation, presence or absence of chlamydospores, macroconidia and microconidia, and reverse mycelium color were used to identify the isolated fungi^20–23^. According to microscopic features such conidial heads, fruiting bodies, degree of sporulation, and the homogeneity traits of conidiogenous cells were examined using the optical light microscope (10 × 90) Olympus CH40. Fungal isolates were grown on malt extract agar medium at 28 °C for 5 days then the cultures were preserved at 4 °C.

#### Molecular identification through 18S rRNA sequencing

##### Genomic DNA extraction

The fungal mycelia were homogenized, and then the DNA extraction solution was added, which included 200 mM Tris–HCl pH 8, 240 mM NaCl, 25 mM EDTA, and 1% SDS. Following the addition of a single volume of phenol/CHCl_3_ in a 1:1 (v/v) ratio and ten minutes of gentle shaking and mixing, the mixture was centrifuged for ten minutes at 15,000 × g. The top phase was moved to a fresh tube, and two volumes of 96% ethanol and 0.1 volumes of 3 M sodium acetate (pH 5.2) were added and thoroughly mixed. The mixture was incubated for 30 min at -20 °C before being centrifuged for 20 min at 15,000 × g (4 °C). After being cleaned with 70% ethanol and let to air dry, the final pellet was re-suspended in 100 µl of sterile bi-distilled water.

##### PCR amplification

The primers ITS1 5’ (TCC GTA GGT GAA CCT GCG G) 3' and ITS4 5’ (TCC TCC GCT TAT TGA TAT GC) 3' were used for the amplification of 18S rRNA gene. An initial denaturation at 95 °C for 2 min and 35 cycles of denaturation at 95 °C for 1 min, annealing at 55 °C for 1 min, extension at 72 °C for 1 min, and final extension for 10 min at 72 °C were the steps taken to carry out the PCR reaction. The amplification products were purified with a multiscreen filter plate (Millipore Corp., Bedford, MA, USA). Utilizing a PRISM Big Dye Terminator v3.1 Cycle sequencing Kit, the sequencing reaction was carried out. Hi-Di formamide (Applied Biosystems, Foster City, CA) was mixed with the DNA samples that included the extension products. After five minutes of incubation at 95 °C and five minutes of cooling on ice, the mixture was examined using an ABI Prism 3730XL DNA analyzer (Applied Biosystems, Foster City, CA).

The fungal 18S rRNA sequences were compared with other gene sequences in the GenBank through the NCBI database (http://www.ncbi.nlm.nih.gov/ BLAST). Phylogenetic tree of isolated fungi in comparison with other registered isolates in the NCBI were constructed and the pairwise sequence combinations were analyzed by the neighbor-joining (NJ) method using Molecular Evolutionary Genetics Analysis (MEGA) software version 4.0 available at http://www.megasoftware.net. The isolated fungal 18S rRNA sequences were deposited at GenBank to assign accession number by NCBI.

### Growth conditions and preparation of fungal biomass and filtrate

Freshly grown cultures on malt extract agar slants were scratched in five milliliters of sterile distilled water to create the inoculum. A 100 ml malt extract broth containing 5 mg/l Cr(VI) final concentration in 500 ml Erlenmeyer flasks was inoculated with 8 × 10^8^ CFU of individual fungi. For five days, they were incubated at 28 °C under shaking at 140 rpm. After passing through Whatman filter paper No. 1 to separate the biomasses, they were rinsed three times with distilled water before being utilized as biosorbent/reductant biomasses as wet weight. On the other hand, the filtrates were utilized for reduction of Cr(VI) to Cr(III). Biomass and filtrates were stored in the freezer until experiments could be conducted on them.

### Colorimetric estimation of Cr(VI)

The diphenyl carbazide method^24^, was used to detect Cr(VI) colorimetrically.

In brief, 1 ml of the culture supernatant or treated aqueous solution after centrifugation was mixed with 9 ml of 0.2 M sulfuric acid and 0.2 ml of 0.25% diphenyl carbazide (in acetone).

The optical density was measured at 540 nm after the reaction mixture was allowed to remain at room temperature for 10 min in order to create a pink-violet complex. The linear regression of the standard graph was used to determine the chromium concentration. The percentage of chromium elimination was calculated using the formula below:

$${\text{Percentage of chromium elimination}}\, = \,\left( {{\mathrm{A}}{-}{\mathrm{B}}} \right)/{\mathrm{A}}\, \times \,{1}00.$$

where A and B are the starting and ending chromium values in milligrams per liter.

### Optimization of growth conditions for the maximum absorption and/or reduction of Cr(VI)

The Effect of growth medium, pH, inoculum size, aeration and chromium concentration were studied according to Mohamed and El-Bendary^25^. All experiments were in triplicates and repeated twice.

#### Effect of growth medium type on Cr(VI) removal

Five mg/l of Cr(VI) were added to the tested media (malt extract, PDB, Czapek Dox, and Sabouraud’s) and inoculated with 8 × 10^8^ CFU of tested fungi separately. These cultures were incubated for 5 days at 28 °C under shaking at 140 rpm. The weight of the biomass and the effectiveness of the biomass and filtrate in removing Cr(VI) were spectrophotometrically measured at 540 nm.

#### Effect of initial medium pH on Cr(VI) removal by tested fungi

Using HCl and NaOH, the pH of the malt extract medium with 5 mg/l of Cr(VI) was varied from pH 2 to pH 9. Malt extract medium (100 ml) at each tested pH was inoculated with 8 × 10^8^ CFU of tested fungi separately and incubated at 28 °C under shaking at 140 rpm for 5 days incubation. Removing Cr(VI) were spectrophotometrically measured at 540 nm.

#### Effect of inoculum size

The effect of different inoculum sizes in the range between (9.5–380 × 10^7^ CFU) on Cr(VI) removal was studied**.** Malt extract medium (100 ml) at pH 2 for *F. verticillioides* or pH 4 for *F. oxysporum* were inoculated with different CFU of tested fungi separately and incubated at 28 °C under shaking at 140 rpm for 5 days incubation.

#### Effect of chromium concentration (in the growth medium) and incubation time on Cr(VI) removal by *F. verticillioides* and *F. oxysporum*

Malt extract medium modified with Cr(VI) at doses of 0–60 mg/l (100 ml) in 500 ml Erlenmeyer flasks were inoculated separately with 38 × 10^8^ CFU of *F. verticillioides* and *F. oxysporum*. The flasks were then incubated for 1–21 days at 28 °C while being shaken at 140 rpm. The removal of Cr(VI) at varying concentrations and intervals was determined spectrophotometrically at 540 nm.

#### Effect of aeration

Two sets of 500 ml Erlenmeyer flasks containing varying quantities (50 to 350 ml) of malt extract medium supplemented with 5 mg/l Cr(VI) were inoculated with 38 × 10^8^ CFU of tested organisms. The first group was incubated under shaking at 140 rpm and 28 °C for 5 days whereas the second set was incubated under static conditions. The effectiveness of mycelia and filtrates in removing Cr(VI) was investigated and determined spectrophotometrically at 540 nm.

### Biosorption and reduction of Cr(VI) in aqueous solution

After growth of tested fungi at optimal conditions (100 ml malt extract broth at pH 2 for *F. verticillioides* or pH 4 for *F. oxysporum*, 5 mg/l Cr(VI), 38 × 10^8^ CFU inoculum, 28 °C incubation temp, shaking at 140 rpm, and 5 days incubation), 1 g wet wight of fungal biomass or 2 ml of fungal filtrate was added to 100 ml of 5 mg/l Cr(VI) solution in 250 ml Erlenmeyer. The mixture was shaken at 140 rpm for 5 days at 28 °C. Cr(VI) removal was determined spectrophotometrically and the concentration of Cr(VI) in the solution was determined as previously mentioned.

### Preparation of the enzyme sources and chromate reductase assay

To investigate enzyme-mediated Cr(VI) reduction, three fungal cell fractions, exo-cell free extract, endo-cell free extract, and membrane debris were separated following the method of Shan et al.^16^. Mycelium pellets were collected by centrifugation at 10,000 rpm for 10 min after incubation of tested fungi under optimal conditions in malt extract broth at 28 °C with shaking at 140 rpm for 5 days. The supernatant obtained was designated as the exo-cell free extract. The mycelium pellets were washed twice, resuspended in 50 mL of 0.1 mM potassium phosphate buffer (pH 7), Shaked at 140 rpm for 30 min, and kept in an ice bath. Cell suspensions were disrupted for 30 min using an ultrasonic unit with 2-s pulses and 4-s intervals. After disruption, the suspension was centrifuged again; the supernatant was collected as the endo-cell free extract, while the precipitate was washed twice, resuspended in 0.1 mM potassium phosphate buffer (pH 7.0) and retained as membrane debris for further analysis.

Chromate reductase activity was determined spectrophotometrically at 540 nm by measuring the reduction of Cr(VI)^26^ using a reaction mixture containing 400 µl of enzyme source (exo-cell free extract, endo-cell free extract, or membrane debris), 200 µl of 0.2 mM NADH, and 200 µl of 0.2 mM K₂Cr₂O₇ in 200 mM phosphate buffer (pH 7). After incubating the mixture at 37 °C for 30 min, the reaction was stopped by adding 500 µl of 20% trichloroacetic acid. Subsequently, 2 ml of 0.5% (w/v) 1,5-diphenylcarbazide in acetone was added to develop a pink color, which was measured at 540 nm. One unit (U) of chromate reductase activity was defined as the amount of enzyme that reduces 1 µM of Cr(VI) per min under these conditions.

### Scanning electron microscopy (SEM)

SEM analysis was carried out to detect morphological changes of the tested fungi after growth under optimum conditions (100 ml malt extract medium at pH 2 for *F. verticillioides* or pH 4 for *F. oxysporum*, 38 × 10^8^ CFU inoculum size, 28 °C incubation temp, shaking at 140 rpm, and 5 days incubation period) with and without 5 mg/l Cr(VI). Fungal cultures without Cr(VI) served as controls. After incubation, fungal mycelia were collected and fixed in 2.5% glutaraldehyde, gold-coated for SEM investigation using JEOL-Model JSM T20 scanning electron microscope, operating at 19 kV.

### Determination of total chromium by atomic absorption spectroscopy AAS

The concentration of total chromium in tested samples was evaluated for both mycelia and filtrates after growth of tested fungi in presence and absence of Cr(VI)^27^. Centrifugation was used to extract the biomass and filtrate from cultures of tested fungi cultivated in 100 ml malt extract medium at pH 2 for *F. verticillioides* or pH 4 for *F. oxysporum*, 5 mg/l Cr(VI), 38 × 10^8^ CFU, 28 °C incubation temp, shaking at 140 rpm, and 5 days incubation. A control samples were obtained after growth of the tested fungi under the same conditions in malt extract medium without Cr(VI). After being cleaned with water, the biomass was dried. The Anton-Paar microwave digestion equipment (Multiwave PRO) was used to microwave digest the samples in an acidic solution. The Agilent 5100 Synchronous Vertical Dual View (SVDV) ICP-OES with Agilent Vapor Generation Accessory VGA 77 was used to determine the metal ions. Each sample was digested to provide a suitable matrix for metal ion measurement and to yield a satisfactory and reliable recovery that was in line with the analytical procedure^28^. A calibration curve for intensity was created for every set of measurements using a blank and three or more Merck Company (Germany) standards. Accuracy and precision of the metal ions measurements were confirmed using external reference standards from Merck, and standard reference material and quality control sample from National Institute of Standards and Technology (NIST).

### Data analysis

The SPSS 16.0 program was used to statistically analyze the collected data using the Duncan’s multiple range test and one-way analysis of variance. The mean value ± standard error was used to express the data. Differences were considered statistically significant if their p-value was < 0.05.

## Results and discussion

Biotechnological applications of microbial green biosorption and enzymatic reduction for the detoxification of Cr(VI) are thought to be an effective green substitute for the chemo-physical removal approach^14^.

In this investigation, two isolates (isolate no. 1 and isolate no. 4) were isolated from 40 mg/l Cr(IV) solution and were recognized both morphologically and molecularly.

### Morphological characteristic of tested fungi

**Fig. 2:**
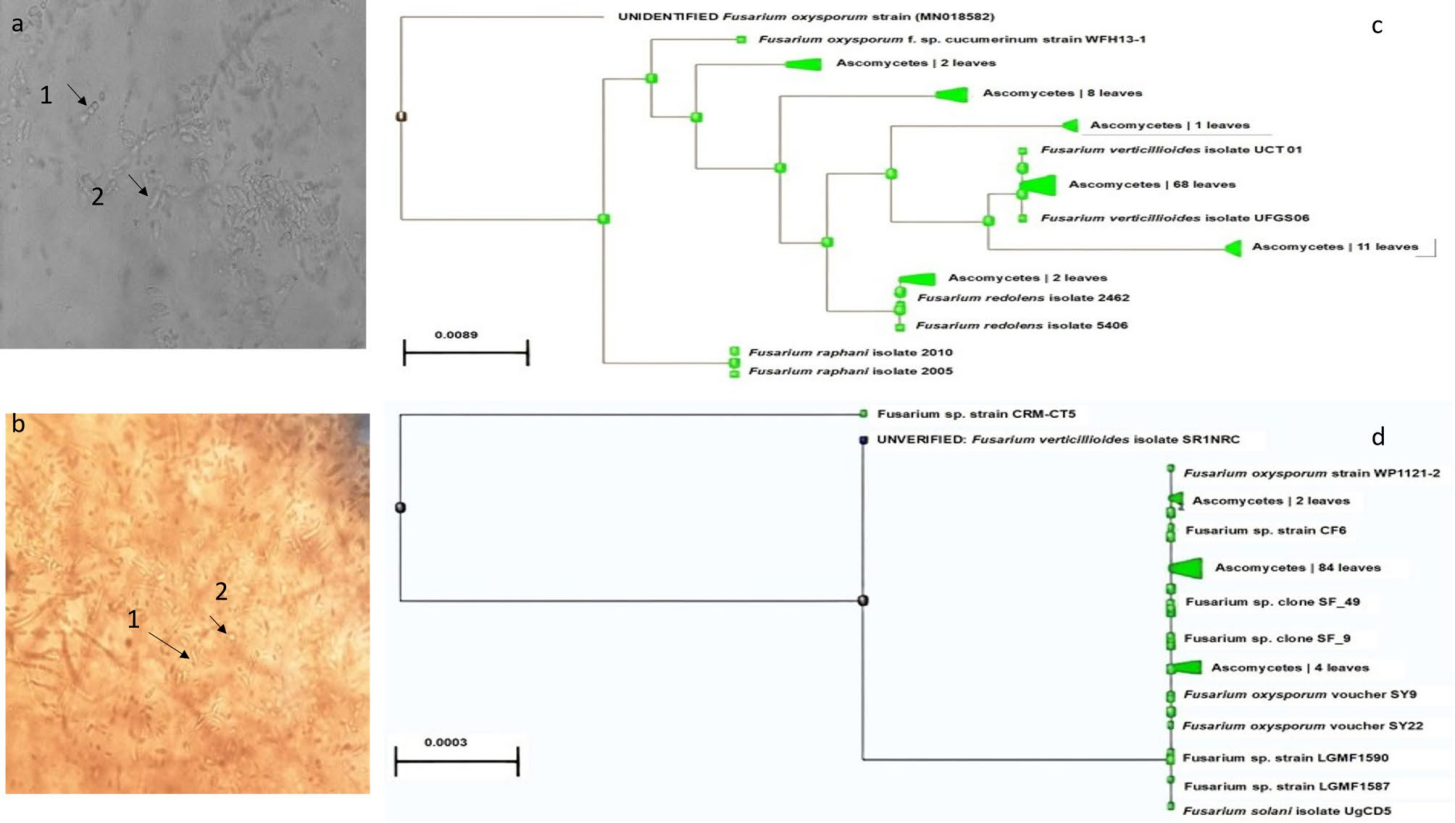
(**a** and **b**) Microscopic features of isolate no 1, *F. verticilloides* and isolate no 4, *F. oxysporium*, respectively, (1) Macrocondia, (2) Microcondia. (**c** and **d**) Phylogenetic tree based on partial 18S rRNA sequence, showing the relationship between isolate no 1 and isolate no 4, respectively with other international species. The tree was constructed using neighbor-joining method.

Isolate no. 1 has white mycelia which develop pink pigments with age. Microconidia formed in long chains and clusters. Microconidia are oval to club-shaped with a flattened base and 0-septate borne from monophialides that may occur in V-shaped pairs to give a rabbit ear appearance. Macroconidia were also observed; however, they were hard to locate and rare. Although chlamydo spores are not generated, enlarged cells in the hyphae could be confused with either pseudochlamydo or chlamydo spores (Fig. 2a).

Isolate no. 4, mycelia looked delicate, ranging from white to pink. Three different kinds of spores were formed by the fungus: chlamydospores, microconidia, and macroconidia. Macroconidia are thin-walled, three to five septate, fusoid-subulate, and pointy at both ends. They are carried on branched conidiophores. Three septate macroconidia are seen in the macroconidia. Microconidia were widely distributed and were carried on simple, laterally growing, oval-ellipsoid, straight-to-curve phialides. Both smooth and rough-walled chlamydospores were prevalent and might form terminally or intercalarily (Fig. 2b).

### Molecular identification of isolated fungi through sequencing of 18S rRNA

The sequence of 1297 bp of 18S rRNA gene from the genomic DNA of isolate no. 1 was found to be closely related to *F. verticillioides* with 99% similarity as shown in Fig. 2c. The 18S rRNA sequence of *F. verticillioides* (Sacc.) was deposited at the GenBank under accession number of ON100819 (https://www.ncbi.nlm.nih.gov/nuccore/ ON100819).

While**,** 1426 bp sequence of 18S rRNA gene from the genomic DNA of isolate no 4 was found to be closely related to *F. oxysporum* with 99% similarity as shown in Fig. 2d. The 18S rRNA sequence of *F. oxysporum* was deposited at the GenBank under accession number of ON010524 (https://www.ncbi.nlm.nih.gov/nuccore/ ON010524).

It was known that the *Fusarium* is a broad genus of filamentous fungi that is extensively found in soil and is often connected with plants. It belongs to a group of fungi known as hyphomycetes^21,22^.

*Fusarium* species have shown strong potential for bioremediation of Cr(VI). *F. proliferatum* S4, isolated from chromium-contaminated soil, can remove up to 100% of Cr(VI) at low concentrations after 12 days^16^. *F. oxysporum* has also demonstrated up to 90% chromium removal efficiency in previous studies^13^. Other *Fusarium* strains such as *F. chlamydosporium* and *F.* sp. MMT1 exhibit high chromium removal capabilities^29^. Additionally, several other fungi including *Aspergillus niger*, *Aspergillus parasiticus*, *Botrytis acladafres*, *Chrysonilia sitophila*, *Pleurotus ostreatus*, *Rhizopus arrhizus*, *Penicillium oxalicum* SL2, *Trichoderma* sp. BSCR02, *Arthrinium malaysianum*, and *Aspergillus terricola* have been reported to efficiently remove Cr(VI) through mechanisms like biosorption and bioreduction^25,29–31^. These fungi offer eco-friendly and effective alternatives for chromium bioremediation in contaminated environments.

### Optimization the growth conditions of fungi for the chromium removal

#### Effect of medium type

**Fig. 3:**
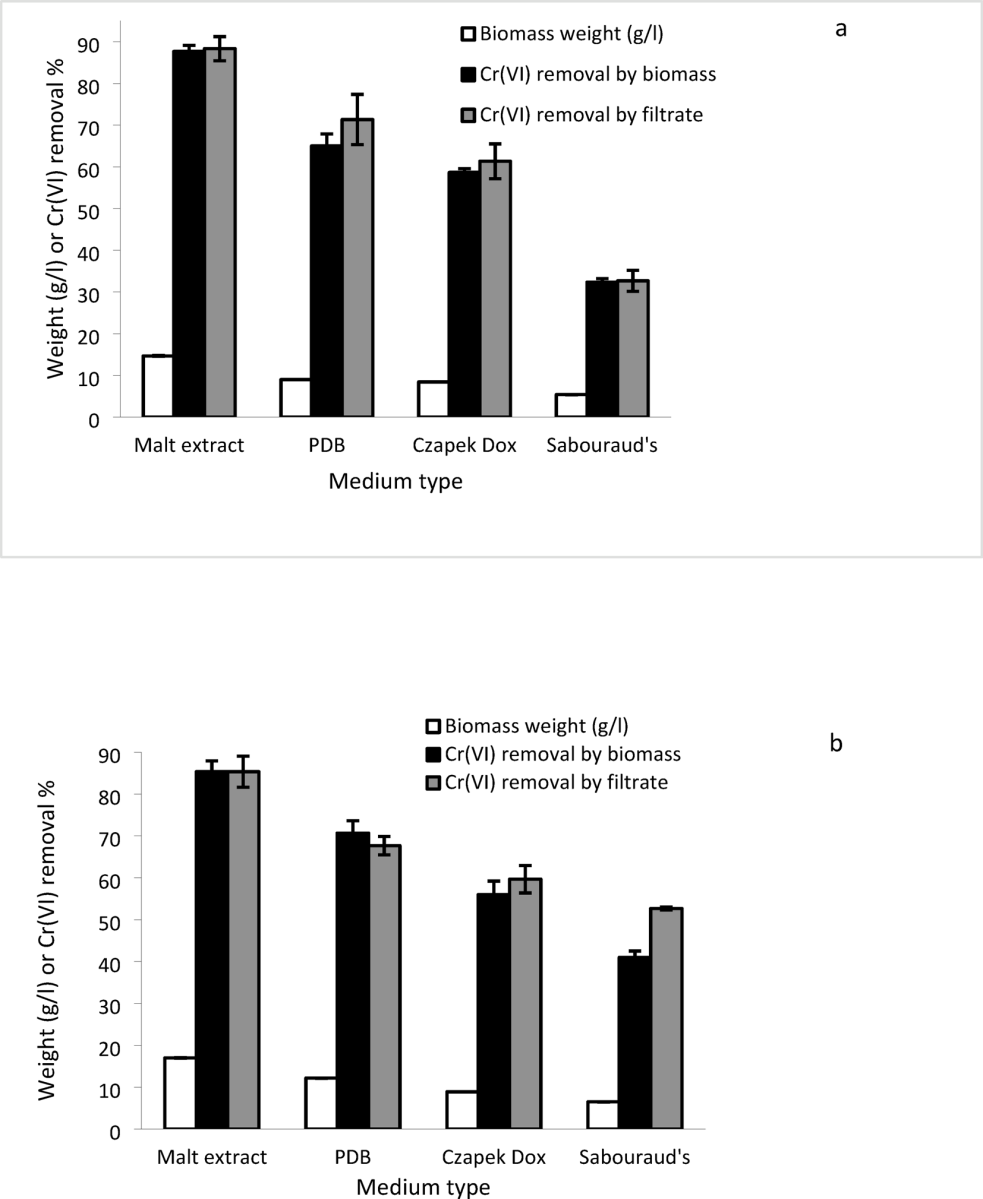
(**a**) Effect of medium type on growth and Cr(VI) removal by *F. verticillioides*. Growth conditions: medium (100 ml), 5 mg/l Cr(VI), 8 × 10^8^ CFU inoculum size, 28 °C incubation temp, shaking at 140 rpm, and 5 days incubation period. (**b**) Effect of medium type on growth and Cr(VI) removal by *F. oxysporium*. Growth conditions: medium (100 ml), 5 mg/l Cr(VI), 8 × 10^8^ CFU inoculum size, 28 °C incubation temp, shaking at 140 rpm, and 5 days incubation period.

Four media were tested for fungal growth, with malt extract medium proving the most effective, yielding the highest biomass wet weights of 15 g/l for *F. verticillioides* and 17 g/l for *F. oxysporum* as shown in Fig. 3 a and b. This medium also achieved the greatest Cr(VI) removal by both fungi, with removal rates between 85 and 88%, making it the preferred choice for fungal cultivation. Growth in malt extract medium increased by approximately 63%, 74%, and 171% for *F. verticillioides* and by 40%, 91%, and 161% for *F. oxysporum* compared to growth in PDA, Czapek Dox, and Sabouraud’s dextrose media, respectively. In contrast, Sabouraud’s medium resulted in the lowest fungal growth and Cr(VI) removal efficiency for both species. A nutrient-rich medium likely enhances mycelial development, increases the number of available biosorption sites, and promotes the production of extracellular metabolites and enzymes involved in chromium reduction. The superior performance of malt extract medium is likely due to its high carbohydrate content (around 90%), especially maltose, along with essential vitamins, amino acids, and salts that support fungal development^32,33^. These findings align with previous reports highlighting malt extract’s rich nutrient profile conducive to fungal growth and bioremediation applications^32^.

#### Effect of pH on chromium removal

pH is a key factor governing Cr(VI) removal because it affects chromium speciation, proton availability, and the ionization of fungal surface functional groups involved in biosorption and reduction^13^.

As shown in Table 1. *F. verticillioides* showed maximum removal at pH 2, achieving 77% and 79% removal for biomass and filtrate, respectively. This agrees with reports showing that strongly acidic conditions enhance the reduction of Cr(VI) to the less toxic Cr(III) form by increasing proton availability and favoring adsorption-reduction processes^34^. *F. oxysporum* exhibited optimal removal at slightly higher acidic pH values ((pH 3–4 for biomass (70%) and pH 4 for filtrate (97%)) indicating species-specific differences in chromium uptake and reductive activity. Notably, the filtrate of *F. oxysporum* maintained consistently high Cr(VI) removal across the tested pH range, declining only to 77% at pH 9.

**Table 1:** Effect of growth medium pH on Cr(VI) removal by tested organism. (Growth conditions: malt extract medium (100 ml), 5 mg/l Cr(VI), 8 × 10^8^ CFU inoculum size, 28 °C incubation temp, shaking at 140 rpm, and 5 days incubation period).

pH	Cr(VI) removal % by			
	*F. verticillioides*		*F. oxysporum*	
	Biomass	Filtrate	Biomass	Filtrate
2	77.4 ± 0.2a	79.4 ± 0.4a	67.3 ± 0.4b	82.7 ± 0.2d
3	66.6 ± 0.4b	69.4 ± 0.3b	69.5 ± 0.2a	94.4 ± 0.5b
4	63.3 ± 0.3c	66.4 ± 0.4c	69.3 ± 0.3a	96.7 ± 0.2a
5	63.2 ± 0.2c	62.5 ± 0.4d	64.6 ± 0.3d	89.7 ± 0.2c
6	55.4 ± 0.4d	61.5 ± 0.3e	65.5 ± 0.4c	89.5 ± 0.5c
7	51.6 ± 0.4e	60.5 ± 0.3f	50.4 ± 0.3e	81.5 ± 0.4e
8	45.4 ± 0.4f	60.1 ± o.2f	50.6 ± 0.4f	80.6 ± 0.3f
9	42.6 ± 0.2 g	55.5 ± 0.4 g	45.5 ± 0.4 g	77 .4 ± 0.4 g

Generally, fungal Cr(VI) removal is favored under acidic conditions, typically between pH 2 and pH 5^35^. The enhanced Cr(VI) removal under acidic conditions can be attributed to the predominance of chromium species that are more readily adsorbed and reduced at low pH^36^. While, at neutral or alkaline pH, chromium becomes less available for interaction with fungal biomass and reducing biosorption efficiency. Similar behavior has been reported for *Paecilomyces lilacinus*, *Aspergillus niger*, and *Trichoderma* spp., which exhibited high chromium tolerance and effective biosorption or bioreduction under acidic conditions, often with optimum performance near pH^37–39^. The optimum pH values observed in the present study were lower than those reported for some other *Fusarium* strains. It was reported that *F.* sp. MMT1 had maximum Cr(VI) biotransformation at pH 5.0 after 72 h^40^. An efficient Cr(VI) reduction by *F. solani* was at pH 5.0 through both intracellular and extracellular mechanisms^41^. It was proposed that low pH promotes protonation of biomass functional groups such as carboxyl, amino, and phosphate groups, thereby increasing electrostatic attraction toward Cr(VI) ions, whereas higher pH reduces this interaction by increasing negative surface charge^13^. In addition, pH strongly influences protein conformation and enzyme activity, which is particularly relevant when reductase-mediated bioreduction contributes to chromium detoxification^42^.

#### Effect of aeration level on Cr(VI) removal under shaking and static conditions

Table 2 shows that maximal Cr(VI) elimination by both *F. verticillioides* and *F. oxysporum* occurred under shaking conditions. For *F. verticillioides*, Cr(VI) removal increased with medium volume from 50 ml (53 and 65%) to 100 ml (87 and 96%) for the biomass and filtrate, respectively then declined with further volume increase. Under static conditions*, F. verticillioides* biomass achieved maximum removal at 100 ml (51%), while filtrate peaked at 50 ml (63%). *F. oxysporum* showed highest removal rates of 68–69% (biomass) and 92% (filtrate) under shaking with 50–100 ml medium, and lower rates under static conditions (50–51% biomass, 68–69% filtrate).

**Table 2:** Effect of different aeration levels on Cr(VI) removal under shaking and static conditions. (Growth conditions: malt extract medium at pH 2 for *F. verticillioides* or pH 4 for *F. oxysporum*), 5 mg/l Cr(VI), 8 × 10^8^ CFU inoculum size, 28 °C incubation temp, shaking at 140 rpm, and 5 days incubation period).

Volume of medium (ml)/flask	CR(VI) removal % of							
	*F. verticillioides* under				*F. oxysporum* under			
	Shaking		Static		Shaking		Static	
	Biomass	Filtrate	Biomass	Filtrate	Biomass	Filtrate	Biomass	Filtrate
50	53 ± 0.2b	65 ± 0.4b	39 ± 0.4c	63 ± 0.5a	69 ± 0.2a	92 ± 0.8a	51 ± 0.2a	69 ± 0.3a
100	87 ± 0.3a	96 ± 0.3a	51 ± 0.3a	60 ± 0.4b	68 ± 0.3b	92 ± 0.3a	50 ± 0.5b	68 ± 0.3b
150	47 ± 0.5c	56 ± 0.3c	45 ± 0.4b	54 ± 0.4c	67 ± 0.2c	89 ± 0.3b	41 ± 0.3c	61 ± 0.5c
200	37 ± 0.5d	52 ± 0.3d	32 ± 0.5d	42 ± 0.5d	56 ± 0.3d	84 ± 0.4c	39 ± 0.2d	51 ± 0.3d
250	33 ± 0.4e	40 ± 0.2e	31 ± 0.5e	33 ± 0.3e	45 ± 0.4e	77 ± 0.3d	34 ± 0.4e	50 ± 0.2e
350	27 ± 0.4f	30 ± 0.4f	22 ± 0.3f	22 ± 0.3f	39 ± 0.3f	71 ± 0.3e	33 ± 0.4f	47 ± 0.4f

Optimal oxygen transfer in shake flask cultures depends critically on the culture volume relative to flask size, as it influences both oxygen diffusion and shear stress. At 100 ml in a 500 ml flask, oxygen transfer is maximized because this volume balances sufficient surface area for gas exchange with adequate mixing, avoiding oxygen limitation while minimizing excessive shear forces. Lower volumes like 50 mL can increase shear stress due to higher agitation impact on a smaller liquid volume, potentially damaging cells or altering metabolism^43^. Conversely, higher volumes (150 mL and above) reduce the gas–liquid interface and increase medium depth, limiting oxygen diffusion to cells and causing hypoxic conditions that impair growth and metabolism. This interplay aligns with findings that oxygen transfer rates depend on hydrodynamic conditions, culture volume, and shaking parameters, which together affect the volumetric mass transfer coefficient and dissolved oxygen availability^44^. Therefore, selecting an optimal culture volume is essential to maintain a balance between adequate oxygen supply and mechanical stress for optimal cell growth and function in aerobic cultures.

It was reported that shaking improves Cr(VI) bioremediation efficiency by fungi through enhanced mass transfer and metabolic activity, but optimal medium volume must be maintained for maximal performance^16,45^.

The bioreduction of Cr(VI) can occur aerobically and anaerobically; in anaerobic environments, electron donors like glutathione and NADH/NADPH cofactors facilitate reductase-driven electron transfer reducing Cr(VI), which can serve as a terminal electron acceptor in respiratory pathways involving cytochromes B and C. In aerobic conditions, Cr(VI) is first reduced to intermediates Cr(V) or Cr(IV), then further to stable Cr(III), completing detoxification^46,47^.

#### Effect of different inoculum size

The efficiency of Cr(VI) reduction by both biomass and filtrates of *F. verticillioides* and *F. oxysporum* increased with inoculum size, rising from approximately 9.5 × 10^7^ to 380 × 10^7^ CFU, reaching removal rates of 80% and 81% for *F. verticillioides* biomass and filtrate, and 84% and 91% for *F. oxysporum*, respectively, as shown in Table 3. Inoculum size is a critical factor influencing microbial growth, enzyme production, and ultimately the effectiveness of bioremediation processes. Larger inoculum sizes provide more fungal biomass, which increases the number of available sorption sites for Cr(VI) ions at a constant contaminant concentration, thereby enhancing biosorption capacity and reduction efficiency. This relationship between inoculum size and Cr(VI) removal has been supported by studies showing that optimized inoculum concentrations improve reduction rates in various microbial systems^16,30^. Increasing inoculum size also promotes faster adaptation and metabolic activity of the microbes, further contributing to improved bioremediation performance. Therefore, controlling inoculum size is essential for maximizing Cr(VI) bioreduction efficiency in fungal treatment systems^25^.

**Table 3:** Effect of different inoculum size on Cr(VI) removal by tested fungi. (Growth conditions: malt extract medium (100 ml) at pH 2 for *F. verticillioides* or pH 4 for *F. oxysporum*, 5 mg/l Cr(VI), 28 °C incubation temp, shaking at 140 rpm, and 5 days incubation period).

Inoculum size (× 10^7^ CFU /ml)	Cr(VI) removal % by			
	*F. verticillioides*		*F. oxysporum*	
	Biomass	Filtrate	Biomass	Filtrate
9.5	16 ± 0.2 g	19 ± 0.3 g	11 ± 0.1 g	21 ± 0.2 g
19	23 ± 0.3f	31 ± 0.4f	21 ± 0.5f	31 ± 0.4f
47	40 ± 0.4e	43 ± 0.4e	33 ± 0.4e	37 ± 0.5e
95	51 ± 0.3d	62 ± 0.4d	37 ± 0.4d	43 ± 0.2d
143	67 ± 0.4c	71 ± 0.4c	56 ± 0.4c	66 ± 0.4c
190	70 ± 0.2b	80 ± 0.5b	73 ± 0.4b	80 ± 0.2b
380	80 ± 0.2a	81 ± 0.1a	84 ± 0.1a	91 ± 0.4a

#### Effect of different potassium dichromate concentrations on the Cr(VI) removal efficiency by *F. verticillioides* and* F. oxysporum*

Tables 4 and 5 indicate that the percentage of Cr(VI) removal decreases as the initial Cr(VI) concentration increases, with no removal observed at 60 mg/L for both *F. verticillioides* and *F. oxysporum*. This decline is likely due to saturation of fungal binding sites and oxidative damage to cells, consistent with findings in *Candida tropicalis* where high Cr(VI) levels impair cell function^48^. The removal efficiency improves significantly with longer incubation periods up to five days for both fungi, stabilizing between 5 and 7 days at lower concentrations (1.25–10 mg/l for *F. verticillioides* and 1.25–40 mg/l for *F. oxysporum*), before gradually decreasing beyond seven days, possibly due to reduced fungal viability or metabolic activity over time^16,48^.

**Table 4:** Effect of Cr(VI) concentration in the medium after growth of *F. verticillioides*. (Growth conditions: malt extract medium (100 ml) at pH 2, 38 × 10^8^ CFU inoculum size, 28 °C incubation temp, shaking at 140 rpm, and 5 days incubation period).

Cr (VI) concentration (mg/l)	Cr(VI) removal % after (days)						
	1	3	5	7	12	17	21
1.25	70 ± 0.4a	89 ± 0.5a	97 ± 0.3a	96 ± 0.5a	80 ± 0.3a	80 ± 0.4a	76 ± 0.3a
2	66 ± 0.3b	82 ± 0.4b	95 ± 0.5b	95 ± 0.3a	80 ± 0.3a	80 ± 0.3a	71 ± 0.4b
5	53 ± 0.3c	70 ± 0.2c	81 ± 0.3c	80 ± 0.1b	67 ± 0.3b	66 ± 0.5b	60 ± 0.4c
10	37 ± 0.5d	50 ± 0.5d	60 ± 0.3d	60 ± 0.4c	51 ± 0.3c	50 ± 0.3c	44 ± 0.3d
20	21 ± 0.3e	29 ± 0.5e	35 ± 0.4e	21 ± 0.4d	22 ± 0.3d	20 ± 0.2d	11 ± 0.5e
40	9 ± 0.3f	10 ± 0.3f	14.4 ± 0.3f	5.3 ± 0.3e	2.7 ± 0.2e	2.4 ± 0.4e	1.3 ± 0.2f
60	0	0	0	0	0	0	0

**Table 5:** Effect of Cr(VI) concentration in the medium after growth of *F. oxysporum*. (Growth conditions: malt extract medium (100 ml) at pH 4, 38 × 10^8^ CFU inoculum size, 28 °C incubation temp, shaking at 140 rpm, and 5 days incubation period).

Cr(VI) concentration (mg/l)	Cr (VI) removal % after (days)						
	1	3	5	7	12	17	21
1.25	78 ± 0.5a	89 ± 0.5a	99 ± 0.5a	98 ± 0.3a	89 ± 0.4a	86 ± 0.4a	80 ± 0.5a
2	77 ± 0.2b	80 ± 0.3b	88 ± 0.3b	87 ± 0.4b	81 ± 0.5b	81 ± 0.4b	76 ± 0.4b
5	69 ± 0.2c	70 ± 0.4c	78 ± 0.2c	78 ± 0.4c	73 ± 0.1c	71 ± 0.5c	66 ± 0.4c
10	51 ± 0.4d	55 ± 0.4d	60 ± 0.5d	60 ± 0.3d	58 ± 0.5d	56 ± 0.4d	45 ± 0.4d
20	39 ± 0.3e	39 ± 0.3e	43 ± 0.4e	43 ± 0.4e	42 ± 0.4e	39 ± 0.1e	31 ± 0.2e
40	10.5 ± 0.5f	11.5 ± 0.4f	14.5 ± 0.4f	14.4 ± 0.4f	11.6 ± 0.2f	10.5 ± 0.3f	8.4 ± 0.4f
60	0	0	0	0	0	0	0

Chromium interacts with fungi through two main mechanisms: extracellular biosorption onto cell walls and intracellular accumulation or complexation with cellular components^49^. Cr(VI) reduction occurs both outside and inside fungal cells, but high Cr(VI) concentrations disrupt gene expression related to chromium uptake and glutathione metabolism, weakening cellular redox balance and increasing toxicity^48,50^. Additionally, Cr(VI) penetrates cell membranes via nonspecific anion carriers, inhibiting enzymes and nucleic acids while generating reactive oxygen species (ROS) that cause oxidative stress, DNA damage, and growth inhibition; however, at lower Cr(VI) levels, fungal antioxidant enzymes such as catalase and superoxide dismutase mitigate ROS damage allowing survival conditions^48,50,51^. These processes highlight the delicate balance between fungal chromium detoxification capacity and toxicity thresholds influencing bioremediation efficiency.

### Chromium reductase activity

Table 6 shows that chromate reductase enzyme activity was detected in the exo- and endo-cell free extracts as well as in fungal membrane debris for both *F. verticillioides* and *F. oxysporum*. The fungal membrane debris exhibited the lowest reductase activity (~ 2.6 U), while the exo-cell free extracts showed about 4 U, and the endo-cell free extracts had slightly higher activities of 4.2 U and 4.4 U for *F. verticillioides* and *F. oxysporum*, respectively. These results indicate that both fungi are promising sources of chromate reductase enzymes capable of reducing toxic Cr(VI) to less harmful Cr(III), a key detoxification mechanism in chromium bioremediation^50,52^. Genomic studies have identified upregulated genes encoding chromate reductases and antioxidant enzymes such as superoxide dismutases under Cr(VI) stress in *Fusarium* species, supporting their enzymatic role in chromium resistance^53^. Microbial Cr(VI) resistance involves enzymatic reduction by chromate reductases, metal immobilization through complex formation with functional groups like hydroxyl, carboxyl, and amino groups, and biosorption or bioaccumulation processes^50,54^. Together, these findings demonstrate that the tested fungi can bioremediate Cr(VI) via combined biosorption and enzymatic reduction pathways, though further research is needed to optimize their application alone or with other agents for treating chromium-contaminated wastewater.

**Table 6:** Chromium reductase activities by exo-cell free extract, endo-cell free extract and fungal membrane debris.

Tested fraction	Chromium reductase activity (U)	
	*F. verticillioides*	*F. oxysporum*
Exo-cell free extract	4.07 ± 0.02	4.03 ± 0.03
Endo- cell free extract	4.20 ± 0.03	4.40 ± 0.07
Fungal membrane debris	2.61 ± 0.03	2.63 ± 0.02

### Cr(VI) reduction in aqueous solution by tested fungi

**Fig. 4:**
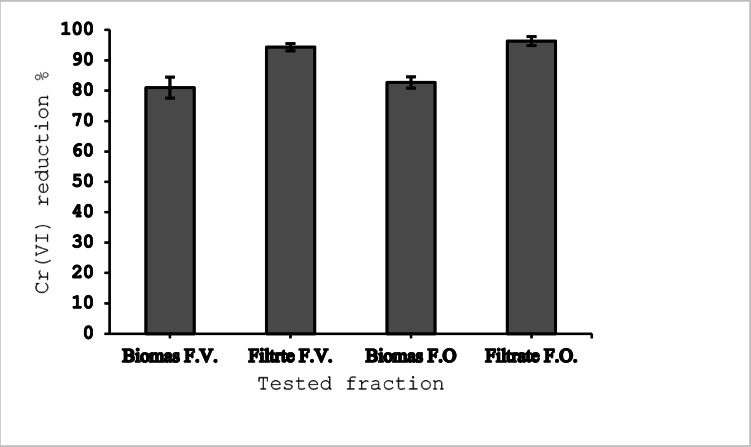
Cr(VI) reduction in aqueous solution by biomass and filtrate of *F. verticillioides* (F.V.) and *F. oxysporium* (F.O.) after grown under optimum conditions (Growth conditions: malt extract medium (100 ml) at pH 2 for *F. verticillioides* or pH 4 for *F. oxysporum*, 5 mg/l Cr(VI), 38 × 10^8^ inoculum size, 28 °C incubation temp, shaking at 140 rpm, and 5 days incubation period).

Under optimized conditions, both the biomass and filtrates of *F. verticillioides* and *F. oxysporum* demonstrated high Cr(VI) reduction efficiencies in aqueous solutions, achieving approximately 81% and 83%for biomass and 94% and 96% for filtrates, respectively (Fig. 4). Total chromium analysis indicated that Cr(VI) biosorption was coupled with a redox reaction releasing proportional amounts of Cr(III) into the supernatant, suggesting a simultaneous biosorption-reduction mechanism^16^. The Cr(VI) reduction by these fungi likely involves two main processes: passive biosorption via chitin-rich cell walls that bind Cr(VI) ions, and active enzymatic reduction mediated by NADH-dependent chromate reductases converting toxic Cr(VI) to less harmful Cr(III)^50,55^. Recent genomic insights reveal that *Fusarium* species possess *chrR* gene clusters encoding chromate reductases, reflecting evolved molecular systems for metal detoxification under chromium stress^53,56^. This dual mechanism enhances fungal potential for effective bioremediation of chromium-contaminated environments.

### Physical characterization of fungal mycelium after Cr(VI) removal

#### Scanning electron microscope (SEM)

**Fig. 5:**
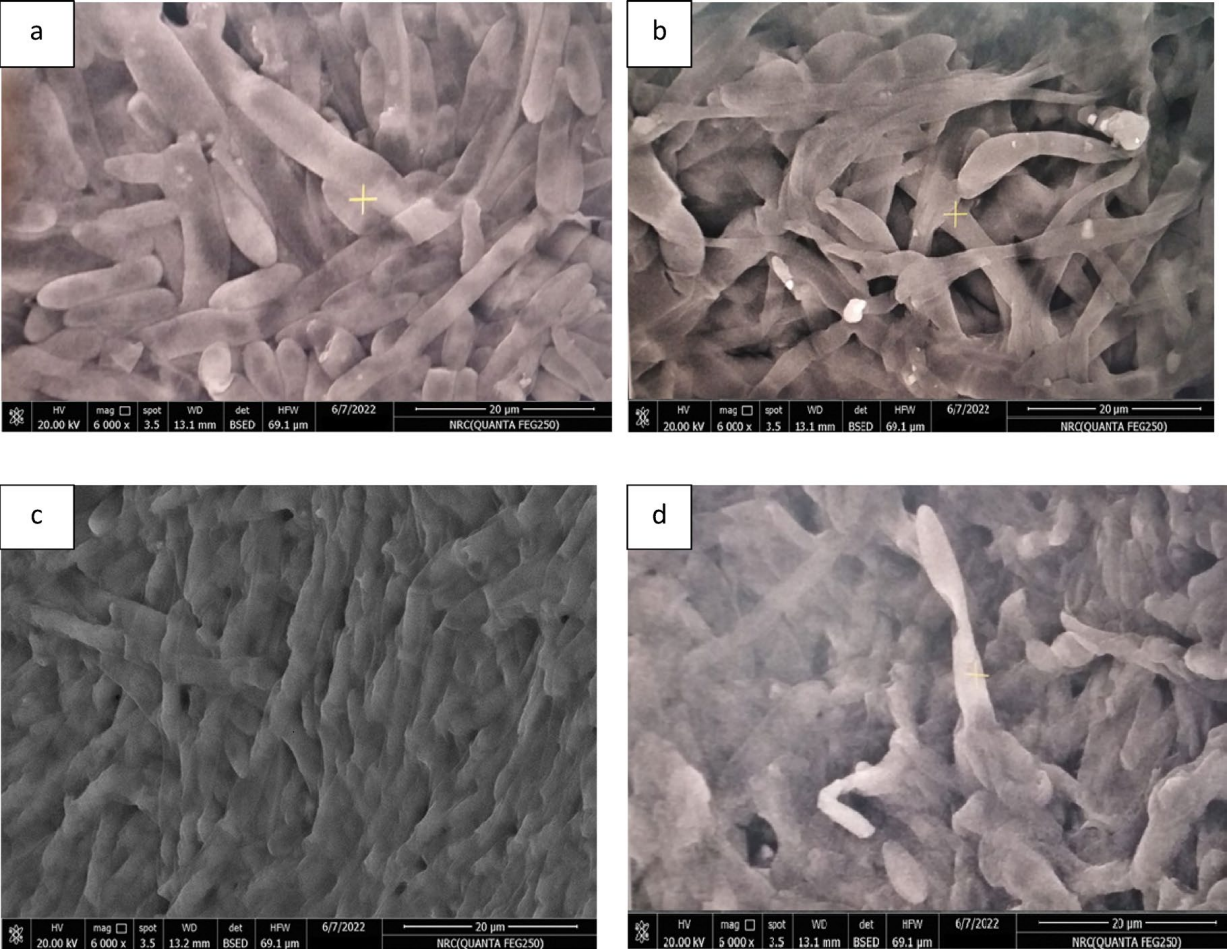
SEM image of (**a**) *F. verticillioides* mycelia grown in control medium without Cr(VI), (**b**) *F. verticillioides* mycelia grown in medium containing Cr(VI), (**c**) *F. oxysporium* mycelia grown in control medium without Cr(VI) and (**d**) *F. oxysporium* mycelia grown in medium containing Cr(VI). Growth conditions: malt extract medium (100 ml) 5 mg/l Cr(VI) at pH 2 for *F. verticillioides* or pH4 for *F. oxysporum*, 38 × 10^8^ CFU inoculum size, 28 °C incubation temp, shaking at 140 rpm, and 5 days incubation period.

Scanning electron micrographs in Figs. 5a–d illustrate the morphology of *F. verticillioides* and *F. oxysporum* mycelia before and after exposure to Cr(VI) stress. In the absence of Cr(VI), both fungi exhibited regular, intact mycelial structures (Fig. 5a and c). Under Cr(VI) stress, *F. verticillioides* showed wrinkled, irregular, and ruptured hyphae with extracellular exudates on the surface (Fig. 5b), while *F. oxysporum* displayed severe hyphal rupture and deterioration (Fig. 5d). These morphological changes reflect adaptive defense mechanisms involving hyphal restructuring and production of extracellular substances, as supported by proteomic studies linking such responses to chromium stress^57^. Extracellular polymeric substances (EPS), including polysaccharides and proteins, are known to chelate Cr(VI) and scavenge ROS, mitigating metal toxicity by forming non-reactive complexes on fungal surfaces^58,59^.

Several *Fusarium* species exhibit morphological changes under Cr(VI) stress similar to wrinkled, ruptured mycelia with extracellular particles. *F. solani* SWUZF-1 shows mycelial swelling, thickening of the cell wall, and ultrastructural alterations including vesicle enlargement when exposed to high Cr(VI) concentrations^60^. *F. proliferatum* S4 demonstrates chromium binding on both cell surfaces and intracellularly, with SEM and TEM revealing involvement of cell surface and internal structures in chromium adsorption and reduction^16^. *F.* sp. MMT1 exhibits significant swelling and formation of cage-like structures on the cell surface under Cr(VI) exposure, along with increased surface roughness detected^40^. Additionally,* F. solani* has been shown to accumulate chromium both intracellularly and extracellularly, with TEM confirming structural changes in hyphae under Cr(VI) stress^41^. These findings confirm that *Fusarium* species respond to Cr(VI) stress with notable morphological alterations including mycelial damage and extracellular particle formation consistent with observations in other fungi.

Several fungi exhibit similar morphological changes under Cr(VI) stress, including wrinkled and ruptured mycelia with extracellular particles. For example, *Macrophomina phaseolina* shows thick-walled, aggregated, branched, short, and broken hyphae with attached irregular particles when exposed to chromium stress^57^. Arbuscular mycorrhizal fungi produce extracellular polymeric substances (EPS) on their surfaces under Cr(VI) stress, which contribute to chromium immobilization and reduction^61^. *Pycnoporus sanguineus* experience oxidative stress leading to apoptosis and mitochondrial dysfunction under Cr(VI), which may relate to structural damage in mycelia^62^. These examples confirm that diverse fungal species respond to Cr(VI) stress with morphological alterations including damaged mycelia and extracellular particle formation.

#### Atomic absorption spectroscopy (AAS)

The stable forms of Cr that are present in the environment are Cr(VI) and Cr(III). Compared to Cr(VI) or total chromium, Cr(III) is more challenging to measure. Hence, most of the studies characterize Cr(VI) reduction by measuring the decrease in Cr(VI) concentration and the production of Cr(III) is indicated by the difference between the total chromium and Cr(VI)^63^.

After 5 days incubation, both tested fungi significantly decreased the Cr(VI) concentration from the initial value of 5 mg/l about 81 and 78% for *F. verticillioides* and *F. oxysporum*, respectively (Tables 4 and 5) as determined by UV–visible spectroscopy. However, there was no discernible change in the overall chromium level of the filtrate or the fungal biomass, indicating that chromium is both adsorbed onto the fungal mycelium and remains in the surrounding medium (Table 7). The high Cr(VI) reduction percentages observed by UV–visible spectroscopy (81% and 78%) for *F. verticillioides* and *F. oxysporum* respectively suggest that enzymatic bioreduction of Cr(VI) to Cr(III) plays a major role alongside biosorption.

**Table 7:** Total chromium in culture filtrate and fungal biomass after 5 days of incubation in medium supplemented with 5 mg/L Cr(VI) as detected by atomic absorption spectroscopy.

Sample	Total chromium
Medium containing 5 mg/L Cr(VI) (without fungi)	5.0 mg/L
Filtrate after growth of *F. verticillioides* in control medium (without chromium)	< 0.01 mg/L
Filtrate after growth of *F. oxysporum* in control medium (without chromium)	< 0.01 mg/L
Biomass of *F. verticillioides* grown in control medium (without chromium)	< 0.01 mg/kg
Biomass of *F. oxysporum* grown in control medium (without chromium)	< 0.01 mg/kg
Filtrate after growth of *F. verticillioides* in medium containing 5 mg/L Cr(VI)	4.7 mg/L
Filtrate after growth of *F. oxysporum* in medium containing 5 mg/L Cr(VI)	5.0 mg/L
Biomass of *F. verticillioides* grown in medium containing 5 mg/L Cr(VI)	5.5 mg/kg
Biomass of *F. oxysporum* grown in medium containing 5 mg/L Cr(VI)	4.98 mg/kg

Growth conditions: malt extract medium (100 ml) at pH 2 for *F. verticillioides* or pH 4 for *F. oxysporum*, 38 × 10^8^ CFU inoculum size, 28 °C incubation temp, shaking at 140 rpm, and 5 days incubation period.

These findings align with genus-level evidence that *Fusarium* spp. can remediate Cr(VI) through a combined cell-wall biosorption and enzymatic bioreduction route. For example, *F. proliferatum* S4 showed substantial Cr(VI) removal with clear contributions from multiple cellular fractions, supporting the concept that detoxification is not limited to adsorption at the biomass surface but extends to intracellular/extracellular redox activity^16^. Similarly, a highly Cr(VI)-resistant *F. solani* strain demonstrated strong Cr(VI) detoxification occurred largely by extracellular processes, reinforcing the practical value of cell-free or secretome-based treatment configurations^60^. Fungi have demonstrated significant potential for chromium removal through various mechanisms including reduction, adsorption, and bioaccumulation. *Aspergillus flavus* CR500 isolated from electroplating wastewater showed up to 89.1% reduction of Cr(VI) to the less toxic Cr(III) within 24 h and also adsorbed chromium on its mycelial surface, effectively detoxifying chromium-contaminated solutions^64^. *Pisolithus* sp1 removed about 99% Cr(VI) mainly via extracellular reduction facilitated by hydrogen ion secretion, showing a dual role of reduction and biosorption^65^. Additionally, *Trichoderma koningiopsis* LBM 253 demonstrated high chromate reductase activity and reduced Cr(VI) toxicity in ecotoxicity tests, confirming its bioremediation potential in contaminated soils^66^. Overall, fungi employ a combination of enzymatic reduction, biosorption, and bioaccumulation processes that enable efficient chromium removal under varied environmental conditions.

The performance of the two isolates in the present study is broadly consistent with previous reports on chromium-remediating fungi, although clear species-level differences were observed. Under the tested conditions, *F. verticillioides* showed better performance at very low pH, whereas *F. oxysporum* exhibited stronger filtrate-mediated Cr(VI) reduction at moderately acidic pH, indicating differences in extracellular versus biomass-associated detoxification behavior. Compared with other *Fusarium* spp., the present isolates showed competitive but not maximal removal efficiency. For example, *F. proliferatum* S4 has been reported to achieve complete removal at low Cr(VI) concentrations after prolonged incubation^16^, while previous studies have also demonstrated strong chromium bioaccumulation and detoxification capacity in *F. oxysporum*^13,49^. In the current work, Cr(VI) reduction in growth medium reached 81% for *F. verticillioides* and 78% for *F. oxysporum* after 5 days, while filtrates under optimized aqueous conditions achieved 94% and 96% removal, respectively. These values indicate that the present isolates are particularly effective in extracellular reduction. Compared with non-*Fusarium* fungi such as *Aspergillus flavus* and *Pisolithus* sp., the tested fungi showed a similar dual mechanism involving biosorption and enzymatic bioreduction^64,65^. Overall, the present isolates should be viewed as efficient and mechanistically well-characterized Cr(VI)-remediating fungi rather than as universally superior performers. However, in agreement with the AAS results, this performance should be interpreted primarily as chromium detoxification through reduction and redistribution rather than complete total chromium removal from the system.

## Conclusion

This study demonstrated that the two chromium-tolerant fungal isolates, identified as *F. verticillioides* and *F. oxysporum*, are capable of Cr(VI) detoxification under acidic conditions through a combined biosorption-bioreduction process. Optimization experiments showed that malt extract medium was the most suitable growth medium for both fungi, while the optimum pH differed between the two species, with *F. verticillioides* performing best at pH 2 and *F. oxysporum* at pH 4. Cr(VI) removal increased with increasing the inoculum size and was favored under shaking conditions, whereas increasing chromium concentration reduced fungal detoxification efficiency.

Both fungal biomass and culture filtrates contributed to Cr(VI) removal, although filtrates generally showed stronger reduction activity, especially in *F. oxysporum*. Chromate reductase activity detected in exo-cellular, endo-cellular, and membrane-associated fractions confirmed that enzymatic reduction contributed to chromium detoxification. In addition, SEM observations revealed clear morphological alterations in fungal hyphae under chromium stress, and AAS results supported that Cr(VI) disappearance was associated mainly with transformation to Cr(III), together with partial chromium association with fungal biomass. Further work should focus on direct Cr(III) quantification, testing in real wastewater, and evaluation of post-treatment steps for total chromium removal.

## Data Availability

The datasets generated and/or analyzed during the current study are available from the corresponding author on reasonable request. The data of 18S rRNA sequencing as the following The 18S rRNA sequence of *F. verticillioides* was deposited at the GenBank under accession number of ON100819 (https://www.ncbi.nlm.nih.gov/nuccore/ON100819). While**,** the 18S rRNA sequence of *F. oxysporum* was deposited at the GenBank under accession number of ON010524 (https://www.ncbi.nlm.nih.gov/nuccore/ ON010524).

## References

[1] Pujalte, E. C. O., Posadas, K. D. B., Morales, T. C. & Chang, A. C. G. Hexavalent chromium [Cr(VI)] tolerance and reduction activity of *Synechococcus* sp. and Synechocystis sp. Isolated in West and South Bay of Laguna de Bay Philippines. *Asian J. Trop. Biotechnol.* **21** (1), 1–9 (2024).

[2] Xia, S. et al. A critical review on bioremediation technologies for Cr(VI)-contaminated soils and wastewater. *Crit. Rev. Environ. Sci. Technol.* **49**, 1027–1078. 10.1080/10643389.2018.1564526 (2019).

[3] Sriharsha, D. V., Kumar, R. L. & Janakiraman, S. Absorption and reduction of chromium by fungi. *Bull. Environ. Contam. Toxicol.* **105**, 645–649 (2020). 32870333 10.1007/s00128-020-02979-7

[4] Hamed, M. T., Elwakil, B. H., Hagar, M., Ghareeb, D. A. & Olama, Z. A. Chromium bioremediation mechanistic action assessment using bacterial consortium isolated from Egyptian petroleum refining company. *Sci. Afr.* **28**, e02642. 10.1016/j.sciaf.2025.e02642 (2025).

[5] Sharma, P., Singh, S. P., Parakh, S. K. & Tong, Y. W. Health hazards of hexavalent chromium (Cr However, temperature and pH have a major impact on protein folding, ionization rate, and enzyme activity since the reductase enzyme is involved in bioreduction activities (VI)) and its microbial reduction. *Bioengineered* **13**, 4923–4938. 10.1080/21655979.2022.2037273 (2022). 35164635 10.1080/21655979.2022.2037273 PMC8973695

[6] Saha, B., Amine, A. & Verpoort, F. Special issue: Hexavalent Chromium: Sources, toxicity, and remediation. *Chem. Africa* **5**, 1779–1780 (2022).

[7] Peng, H. et al. Reduction behavior of chromium (VI) with oxalic acid in aqueous solution. *Preprints* **202008**(0427), v1 (2020). 10.1038/s41598-020-74928-7 PMC7575598 33082489

[8] Ren, B. et al. Freezing/thawing pretreatment of dormant *Aspergillus niger* spores to increase the Cr (VI) adsorption capacity: Process and mechanism. *RSC Adv.* **11**, 7704–7712. 10.1039/D0RA10198B (2021). 35423259 10.1039/d0ra10198b PMC8695046

[9] Yan, X. et al. Lab-scale evaluation of the microbial bioremediation of Cr(VI): Contributions of biosorption, bioreduction, and biomineralization. *Environ. Sci. Pollut. Res.* **28**, 22359–22371. 10.1007/s11356-020-11852-3 (2021). 10.1007/s11356-020-11852-3 33417128

[10] Pandey, K., Saharan, B. S., Kumar, R., Jabborova, D. & Duhan, J. S. Modern-day green strategies for the removal of chromium from wastewater. *J. Xenobiot.*10.3390/jox14040089 (2024). 39584954 10.3390/jox14040089 PMC11587030

[11] Adeniran, O. & Shugaba, A. Bioremediation of hexavalent chromium in potassium dichromate solution by *Botrytis aclada fres* and *Chrysonilia sitophila*. *Int. J. Biotechnol. Wellness Ind.* **2016**(5), 32–38 (2016).

[12] Aslam, A., Kanwal, F., Javied, S., Nisar, N. & Torriero, A. A. J. Microbial biosorption: A sustainable approach for metal removal and environmental remediation. *Int. J. Environ. Sci. Technol.*10.1007/s13762-025-06511-1 (2025).

[13] Tang, X. et al. Study on detoxification and removal mechanisms of hexavalent chromium by microorganisms. *Ecotoxicol. Environ. Saf.* **208**, 111699. 10.1016/j.ecoenv.2020.111699 (2021). 33396030 10.1016/j.ecoenv.2020.111699

[14] Bhattacharya, A., Gupta, A., Kaur, A. & Malik, D. Alleviation of hexavalent chromium by using microorganisms: Insightinto the strategies and complications. *Water Sci. Technol.* **79**, 411–424 (2019). 30924796 10.2166/wst.2019.060

[15] Sule, L. O., Ogunlana, K. S., Oluwafemi, O. C. & Adebesin, I. O. Heavy metal tolerance of fungal and bacterial isolates, and their functional groups following biosorption. *Ceylon J. Sci.* **52**(2), 143–153. 10.4038/cjs.v52i2.798 (2023).

[16] Shan, B. et al. Hexavalent chromium reduction and bioremediation potential of *Fusarium proliferatum* S4 isolated from chromium-contaminated soil. *Environ. Sci. Pollut. Res.* **29**, 78292–78302. 10.1007/s11356-022-21323-6 (2022). 10.1007/s11356-022-21323-6 35690705

[17] Baldiris, R., Acosta-Tapia, N., Montes, A., Hernández, J. & Vivas-Reyes, R. Reduction of hexavalent chromium and detection of chromate reductase (Chirr) in *Stenotrophomonas maltophilia*. *Molecules* **23**, 406. 10.3390/molecules23020406 (2018). 29438314 10.3390/molecules23020406 PMC6017488

[18] Chatterjee, S. & Das, S. Whole-genome sequencing of biofilm-forming and chromium-resistant mangrove fungus *Aspergillus niger* BSC-1. *World J. Microbiol. Biotechnol.* **39**(2), 55. 10.1007/s11274-022-03476-0 (2023). 10.1007/s11274-022-03497-w 36565384

[19] Wang, X. et al. Investigation on mechanism of hexavalent chromium bioreduction by *Escherichia* sp. TH-1 and the stability of reduction products. *J. Environ. Chem. Eng.* **10**, 107231. 10.1016/j.jece.2022.107231 (2022).

[20] Barron, G. L. *The Genera of Hyphomycetes from Soil* (Williams and Wilkins Co., 1968).

[21] Booth, C. The genus *Fusarium*. Commonwealth Mycological Institute, Kew, Surrey, England (1971).

[22] Booth, C. *Fusarium Laboratory Guide to the Identification of Major Species* (Common wealth Mycological Institute, 1977).

[23] Ainsworth, G. C. *Ainsworth and Bisby’s Dictionary of the Fungi* (Commonwealth Mycological Institute, 1971).

[24] Sugasini, A. & Rajagopal, K. Hexavalent chromium removal from aqueous solution using *Trichoderma viride*. *Int. J. Pharma. Biosci.* **6**, 485–495 (2015).

[25] Mohamed, L. A. & El-Bendary, M. A. Hexavalent chromium removal by fungal adsorbent, *Aspergillus terricola*. *J. Global Ecol. Environ.* **13**, 49–57 (2021).

[26] Mohamed, M. S. M., El-Arabi, N. I., El-Hussein, A., El-Maaty, S. A. & Abdelhadi, A. A. Reduction of chromium-VI by chromium-resistant *Escherichia coli* FACU: A prospective bacterium for bioremediation. *Folia Microbiol (Praha)* **65**, 687–696. 10.1007/s12223-020-00771-y (2020). 31989423 10.1007/s12223-020-00771-y

[27] Cardenas-Gonzalez, J. F. & Acost-Rodrıguez, I. Hexavalent chromium removal by a Paecilomyces sp. Fungal strain isolated from environment. *Bioinorg. Chem. Appl.* **2010**, 676243. 10.1155/2010/676243 (2010). 20634988 10.1155/2010/676243 PMC2902107

[28] APHA (American Public Health Association), AWWA (American Water Works Association), and WEF (Water Environment Federation). (2021). Standard Methods for the Examination of Water and Wastewater, 24^th^ edition. (Lipps, W.C., Baxter, T.E., Braun-Howland, E.B.

[29] García-Hernández, M., Villarreal-Chiu, J. & Garza-González, M. Metallophilic fungi research: An alternative for its use in the bioremediation of hexavalent chromium. *Int. J. Environ. Sci. Technol.* **14**, 2023–2038. 10.1007/s13762-017-1348-5 (2017).

[30] Bibi, S. et al. Bioremediation of hexavalent chromium by endophytic fungi; safe and improved production of *Lactuca sativa* L. *Chemosphere* **211**, 653–663. 10.1016/j.chemosphere.2018.07.197 (2018). 30098561 10.1016/j.chemosphere.2018.07.197

[31] Dubey, P., Farooqui, A., Patel, A. & Srivastava, P. Microbial innovations in chromium remediation: Mechanistic insights and diverse applications. *World J. Microbiol. Biotechnol.*10.1007/s11274-024-03936-w (2024). 38553582 10.1007/s11274-024-03936-w

[32] Acharya, T. & Hare, J. Sabouraud agar and other fungal growth media. *Fungal Biol.*10.1007/978-3-030-83749-5_2 (2022).36008050

[33] Ahmad, L., Albiski, F., Murshed, R., Mando, H. & Okla, B. Effect of different nutrient media on mycelial growth of different species of medicinal mushroom. *Arab. Am. Univer. J.*10.35517/aaup-2025.v11.1.07 (2025).

[34] Morales-Pontet, N., Fernández, C. & Botté, S. Chromium removal by microbial mats: Understanding the effect of salinity and pH. *Environ. Monit. Assess.*10.1007/s10661-024-12847-0 (2024). 38958830 10.1007/s10661-024-12847-0

[35] Mukhtar, H., Hina, I., Muneer, B., Gohar, U. & Akhtar, M. Kinetics of the biosorptive removal of chromium from water using mycelial biomass of *Aspergillus oryzae*. *Bioremediat. J.* **28**, 99–109. 10.1080/10889868.2022.2136133 (2022).

[36] Pradhan, D., Sukla, L., Mishra, B. & Devi, N. Biosorption for removal of hexavalent chromium using microalgae *Scenedesmus* sp.. *J. Clean. Prod.*10.1016/j.jclepro.2018.10.288 (2019).

[37] Cyriac, S., John, N. & Daniel, E. Biosorption of hexavalent chromium using fungal strains isolated from soil. *Kristu**Kristu Jayanti J. Core Appl. Biol.*10.59176/kjcab.v2i1.2251 (2022).

[38] Tello-Galarreta, F. et al. In vitro effect of molasses concentration, pH, and time on chromium removal by *Trichoderma* spp. from the effluents of a Peruvian tannery. *Processes*10.3390/pr11051557 (2023).

[39] Gizaw, B., Alemu, T., Ebsa, G. & Tsegaye, Z. Fungi species identified from polluted environment for chromium sequestration and solid state fermentation on tannery shaving waste. *Biocatal. Agric. Biotechnol.*10.1016/j.bcab.2024.103352 (2024).

[40] Guria, M., Guha, A. & Bhattacharyya, M. A green chemical approach for biotransformation of Cr(VI) to Cr(III), utilizing *Fusarium* sp. MMT1 and consequent structural alteration of cell morphology. *J. Environ. Chem. Eng.* **2**, 424–433. 10.1016/j.jece.2014.01.016 (2014).

[41] Sen, M., Dastidar, M. G. & Roychoudhury, P. K. Hexavalent chromium reduction and its distribution in the Sen cell and medium by chromium resistant *Fusarium solani*. *Int. J. Eng. Technol. Innov.* **3**, 01–09 (2013).

[42] Zhang, K. D. & Li, F. L. Isolation and characterization of a chromium-resistant bacterium *Serratia* sp. Cr-10 from a chromate-contaminated site. *Appl. Microbiol. Biotechnol.* **90**, 1163–1169 (2011). 21318365 10.1007/s00253-011-3120-y

[43] Cerrone, F. & O’Connor, K. Cultivation of filamentous fungi in airlift bioreactors: advantages and disadvantages. *Appl. Microbiol. Biotechnol.*10.1007/s00253-025-13422-4 (2025). 39928147 10.1007/s00253-025-13422-4 PMC11811475

[44] Buchelly, R. et al. Volumetric oxygen transfer coefficient effect on biomass, bioactive compounds production, and kinetic behavior of G. lucidum in submerged culture using a complex medium. *Braz. Arch. Biol. Technol.*10.1590/1678-4324-2022210618 (2022).

[45] Espinoza-Sánchez, M., Arévalo-Niño, K., Quintero-Zapata, I., Castro-González, I. & Almaguer-Cantú, V. Cr(VI) adsorption from aqueous solution by fungal bioremediation based using *Rhizopus* sp. *J. Environ. Manage.* **251**, 109595. 10.1016/j.jenvman.2019.109595 (2019). 31561145 10.1016/j.jenvman.2019.109595

[46] Karimi-Maleh, H. et al. Recent advances in removal techniques of Cr(VI) toxic ion from aqueous solution: A comprehensive review. *J. Mol. Liq.*10.1016/j.molliq.2020.115062 (2020).

[47] Ramli, N. et al. A review of the treatment technologies for hexavalent chromium contaminated water. *Biometals* **36**, 1189–1219. 10.1007/s10534-023-00512-x (2023). 37209220 10.1007/s10534-023-00512-x

[48] Poljšak, B., Pócsi, I., Raspor, P. & Pesti, M. Interference of chromium with biological systems in yeasts and fungi: A review. *J. Basic Microbiol.*10.1002/jobm.200900170 (2010). 19810050 10.1002/jobm.200900170

[49] Khurshid, S., Shoaib, A., Javaid, A. & Abid, K. Bioaccumulation of chromium by *Fusarium oxysporum*. *ScienceAsia* **42**, 92–98. 10.2306/scienceasia1513-1874.2016.42.092 (2016).

[50] Gu, Y. et al. Mechanism of Cr(VI) reduction by *Aspergillus niger*: Enzymatic characteristic, oxidative stress response, and reduction product. *Environ. Sci. Pollut. Res.* **22**, 6271–6279. 10.1007/s11356-014-3856-x (2015). 10.1007/s11356-014-3856-x 25408081

[51] Ahammed, G., Shamsy, R., Liu, A. & Chen, S. Arbuscular mycorrhizal fungi-induced tolerance to chromium stress in plants. *Environ. Pollut.*10.1016/j.envpol.2023.121597 (2023). 37031849 10.1016/j.envpol.2023.121597

[52] Mohammed, S. et al. Kinetic studies and bioremediation potential of chromate reductase-characterized *Aspergillus flavus* from tannery effluent. *Niger. J. Biochem. Mol. Biol.*10.4314/njbmb.v38i4.2 (2023).

[53] Chen, A. et al. Genetic mapping, candidate gene identification and marker validation for host plant resistance to the race 4 of *Fusarium oxysporum* f. sp. *cubense* using *Musa acuminata* ssp. *malaccensis*. *Pathogens* **12** (6), 820. 10.3390/pathogens12060820 (2023). PMID: 37375510; PMCID: PMC10303076. 10.3390/pathogens12060820 PMC10303076 37375510

[54] Mala, J., Takeuchi, S., Sujatha, D., & Mani, U. Microbial Chromate Reductases: Novel and Potent Mediators in Chromium Bioremediation-A Review. 32–44. 10.37256/amtt.112020222 (2020).

[55] González, J. F. C. et al. Biotransformation of chromium (VI) via a reductant activity from the fungal strain *Purpureocillium lilacinum*. *J Fungi (Basel)* **7**(12), 1022. 10.3390/jof7121022 (2021). 34947004 10.3390/jof7121022 PMC8707924

[56] Melati, I., Rahayu, G., S., Effendi, H., Henny, C., & Susanti, E. Chromium (VI) bioremediation potential of dark septate endophytic (DSE) fungi. IOP Conference Series: Earth and Environmental Science, 1201. 10.1088/1755-1315/1201/1/012077 (2023).

[57] Shoaib, A., Nisar, Z. & JavaidJaved, N. A. S. Necrotrophic fungus *Macrophomina phaseolina* tolerates chromium stress through regulating antioxidant enzymes and genes expression (MSN1 and MT). *Environ. Sci. Pollut. Res.* **26**, 12446–12458. 10.1007/s11356-019-04457-y (2019). 10.1007/s11356-019-04457-y 30847809

[58] Zadeh, P. H. et al. Impacts of metal stress on extracellular microbial products, and potential for selective metal recovery. *Ecotoxicol. Environ. Saf.* **252**, 114604. 10.1016/j.ecoenv.2023.114604 (2023). 36758509 10.1016/j.ecoenv.2023.114604

[59] Kumar, S. & Singh, M. Therapeutic strategies for chromium-induced neurotoxicity: Exploiting NPTX2 and autophagy pathways in CNS cells institute of pharmaceutical research. In *Proc. Presented at the 3rd International Electronic Conference on Biomolecules*, Vol. 103 (1), p. 22 (GLA University, Mathura 281406, India, 2024). 10.3390/proceedings2024103022.

[60] Tuo, W., Zuo, S. & Dong, J. Detoxification of Cr(Ⅵ) and extracellular formation of nanoparticles Cr_2_O_3_ by a highly Cr(Ⅵ)-resistant fungus *Fusarium solani* SWUZF1. *Environ. Technol. Innov.*10.1016/j.eti.2023.103377 (2023).

[61] Wu, S. et al. Chromium immobilization by extra- and intraradical fungal structures of arbuscular mycorrhizal symbioses. *J. Hazard. Mater.* **316**, 34–42. 10.1016/j.jhazmat.2016.05.017 (2016). 27209517 10.1016/j.jhazmat.2016.05.017

[62] Feng, M. et al. Hexavalent chromium induced oxidative stress and apoptosis in *Pycnoporus sanguineus*. *Environ. Pollut.* **228**, 128–139. 10.1016/j.envpol.2017.05.012 (2017). 28528260 10.1016/j.envpol.2017.05.012

[63] Zhong, L. et al. Nitrate effects on chromate reduction in a methane-based biofilm. *Water Res.* **115**, 130–137. 10.1016/j.watres.2017.03 (2017). 28273443 10.1016/j.watres.2017.03.003

[64] Kumar, V. & Dwivedi, S. Hexavalent chromium reduction ability and bioremediation potential of *Aspergillus flavus* CR500 isolated from electroplating wastewater. *Chemosphere* **237**, 124567. 10.1016/j.chemosphere.2019.124567 (2019). 31549665 10.1016/j.chemosphere.2019.124567

[65] Shi, L. et al. Hydrogen ions and organic acids secreted by ectomycorrhizal fungi, *Pisolithus* sp1, are involved in the efficient removal of hexavalent chromium from waste water. *Ecotoxicol. Environ. Saf.* **161**, 430–436. 10.1016/j.ecoenv.2018.06.004 (2018). 29908454 10.1016/j.ecoenv.2018.06.004

[66] Tatarin, A., Aranguiz, C., Sadañoski, M., Polti, M. & Fonseca, M. Fungal species originating from chromium contaminated soil for ecofriendly and biotechnological processes. *Appl. Soil Ecol.*10.1016/j.apsoil.2023.105231 (2023).

